# A Digital Architecture for the Real-Time Tracking of Wearing off Phenomenon in Parkinson’s Disease

**DOI:** 10.3390/s22249753

**Published:** 2022-12-13

**Authors:** Giovanni Mezzina, Daniela De Venuto

**Affiliations:** Department of Electrical and Information Engineering, Politecnico di Bari, 70125 Bari, Italy

**Keywords:** EEG, EMG, heterogeneous computing platform, wearing off, Parkinson’s Disease

## Abstract

Levodopa administration is currently the most common treatment to alleviate Parkinson’s Disease (PD) symptoms. Nevertheless, prolonged use of Levodopa leads to a wearing-off (WO) phenomenon, causing symptoms to reappear. To build a personalized treatment plan aiming to manage PD and its symptoms effectively, there is a need for a technological system able to continuously and objectively assess the WO phenomenon during daily life. In this context, this paper proposes a WO tracker able to exploit neuromuscular data acquired by a dedicated wireless sensor network to discriminate between a Levodopa benefit phase and the reappearance of symptoms. The proposed architecture has been implemented on a heterogeneous computing platform, that statistically analyzes neural and muscular features to identify the best set of features to train the classifier model. Eight models among shallow and deep learning approaches are analyzed in terms of performance, timing and complexity metrics to identify the best inference engine. Experimental results on five subjects experiencing WO, showed that, in the best case, the proposed WO tracker can achieve an accuracy of ~84%, providing the inference in less than 41 ms. It is possible by employing a simple fully-connected neural network with 1 hidden layer and 32 units.

## 1. Introduction

Parkinson’s disease (PD) is a progressive disorder of the nervous system, which is related to the reduction of dopamine production in brain cells. It translates to some characteristic motor symptoms such as muscle stiffness, tremors, balance, gait, speech and sleep disorders [1]. To address the PD-related dopamine reduction and relief of PD symptoms, currently, one of the most common approaches is the Levodopa (L-dopa) administration [1,2].

Nevertheless, within a few years from diagnosis, the therapeutic effect of L-dopa starts shortening. In this situation, there is a clear fluctuation between L-dopa benefits immediately after the administration (ON phase), and their disappearance (OFF phase). This phenomenon is known as Wearing Off (WO) [2].

Typically, WO starts occurring after 2 years from the diagnosis, but within 5 years about 40% of PD patients experience this phenomenon [3].

Currently, the standard for WO identification consists of an ambulatorial visit, and a report provided by the patient, by using self-rated diaries and clinical interviews [3,4]. This approach shows some shortcomings because most of the non-demented PD patients (30–50% [3]) demonstrated to be not self-aware of motor complications. Moreover, up to 25% of WO cases remain undetected [3,5].

Identifying the rising of the WO phenomenon, as well as its specific features, is crucial to allow a timely intervention in treatment by adapting it for an improvement in the quality of life.

In this context, the present paper proposes a WO tracker, which analyzes neuromuscular data, wirelessly gathered during gait, to provide an objective measurement employable as support during clinical assessment.

The proposed WO detector employs a heterogeneous computing platform, realized, and described in our previous work [6]. The platform in [6], born to assess cortico-muscular implications in PD stratification and loss of balance risk assessment, has been here exploited to identify significant features to discriminate between the ON and OFF phases of the WO phenomenon. Experimental results on five subjects experiencing WO showed that the proposed system is able to discriminate between the two WO phases with an accuracy of 83.72% by exploiting a simple dense neural network with a single hidden layer of 32 units/layer. The low complexity of the presented architecture makes the proposed model suitable for most of the embedded platforms currently available at low-cost. The system inference is ensured, in the worst case, in ~44 ms after each walking step, paving the way to real-life scenarios applicability of the proposed digital architecture.

The paper is organized as follows. Section 2 investigates the current technological solutions for WO tracking. Section 3 outlines the proposed digital architecture. Section 4 presents and discusses the experimental results,

## 2. Related Works

To date, several technological tools have been introduced to assess the symptoms, stratification, and neuromuscular implications of PD [7,8,9,10,11,12], and many of these technologies rely on gait and posture analysis. In this context, it is possible to find solutions that employ video system for gait monitoring as reported by Cimolin et al. in [7], which propose a non-invasive solution, based on a single camera to estimate specific features of gait patterns of PD on a reduced walking path compatible with domestic settings. The proposed platform was demonstrated to be useful in accurately investigating bradykinesia, rest tremor, rigidity, and postural instability. However, the proposed solution would require all environments involved in the patient’s daily routine activities to be equipped with a set of dedicated cameras and a gateway. Solutions that take advantage of wearable sensors are also proposed at state-of-the-art for the same purpose. One example is the system implemented by Zhang et al. in [11], which consists of a wireless gait monitoring system detecting plantar bend and impact force to assess various gait indices such as asymmetry and stride cycle length. The gait monitoring and analysis technological solutions can employ motion and physiological sensors, as per the device proposed in [12]. To this aim, gyroscopes and surface electromyography have been embedded in wearable devices that sense simultaneous movements and action potentials of antagonist leg muscles to detect Freezing of Gait (FOG) and to automatically discriminate the FOG phenotypes.

Many of the above solutions, however cutting-edge, are geared toward identifying general neuromuscular implications or symptoms related to PD. However, only a few state-of-the-art solutions [13,14,15,16,17,18] ensure the use of technologies aimed at providing real-time WO detection and objective measurement of the specific phenomenon in PD, which constitute the focus of the present paper.

A possible solution has been proposed by Victorino et al. in [13]. They propose the use of a smart band Garmin vivosmart4 fitness tracker to extract the built-in heart rate, step cadence, stress score, and a sleep classification parameter to discriminate between the ON and OFF phases of WO. However, the statistical analysis conducted on 2 PD patients led the authors to conclude that the monitored parameters are subjectively different, and, for this reason, an individual-level analysis should be conducted to develop more general classification models. Moreover, although the manuscript provides an interesting analysis of the statistical significance of the analyzed parameters by emphasizing the differences in the ON state and OFF state, it does not implement a WO detection step based on a classification model. It is, therefore, not possible to define the system’s performance metrics.

Another solution in the WO detection framework, is the one proposed by Ijima et al. in [14]. Authors in [14] focus on the periodicity of walking in patients with PD, equipped with a belt embedding an accelerometer. Acceleration time series acquired during walking are analyzed through a Long Short-Term Memory (LSTM) network, that extracts the error between the current series and the ON state one. The model calculates the Mahalanobis distance between them to examine anomalies. Anomalies in this distance can be used to identify the WO. Although the authors propose a new set of features with prospective potential, they do not evaluate the actual impact on a classification system. Objective metrics about the detection of the WO phenomenon cannot be extracted from [14]. Another solution in the real-time WO detection context is provided by Pierce et al. [15]. Patients have been equipped with a smartphone attached to the right side of the pelvis using a common smartphone belt pouch. Triaxial accelerometer and gyroscope data from the smartphone are used to identify dysfunctional gait patterns. The extracted features have been employed to train generalized linear models with regularization (RGLM), neural networks (NN), and random forest (RF) classification models. Across all subjects, RGLM, NN and RF achieved accuracies of 72.4%, 80.2% and 86.8% respectively [15].

Moreover, medical fluctuations due to L-dopa have been also investigated by [16]. The authors acquired gyroscope data via two wearable inertial measurement units (IMU). Gathered time series are then analyzed offline via Matlab^®^ to extract 40 segment-based features and 22 time-frequency domain ones. The system demonstrated to be able in discriminating between ON and OFF states with an accuracy of 83.56% in the best case (Hierarchical features). Despite the proposed approach providing an interesting overview of several feature extraction methods, the main drawback of the proposed solution lies in the high computation load of this stage, which forced the authors in operating offline via Matlab^®^.

Two IMUs mounted on the insteps of patients’ feet have been also employed by Moradi et al. in [17] to detect the WO phenomenon. Each IMU, embedding a 3D-accelerometers and a 3D-gyroscopes, has been used to evaluate three temporal and eight spatial features from each stride on both feet. A Support Vector Machine (SVM) and an RF model have been extracted to test the feature set. RF returns the best performance by achieving the 77% of accuracy.

Kilinçalp et al. in [18] analyzed the need to obtain objective measures for the WO phenomenon detection and the management of L-dopa intestinal gel infusion. Specifically, the analysis concerns the use of a device widely employed in clinical practice, the Parkinson’s KinetiGraph (PKG by Global Kinetics Corporation^TM^, Australia) [9,12,19,20]. PKG consists of a smartwatch that monitors motions during activities of daily living. At the end of the recording period, data are uploaded to a computer for offline processing. The PKG provides scores for dyskinesia and bradykinesia to identify WO-related motor fluctuations, immobility, and tremor. The authors’ experience with using the PKG suggests that motor fluctuations are detected roughly in 50–60% of cases.

Despite its use for WO assessment in the clinical framework, as demonstrated by [3], PKG operates offline after long periods of recording. It excludes the possibility of operating dynamic change in L-dopa treatments in the dependence of a real-time analysis. Moreover, it is a smartwatch and, thus, as wrist-worn device, PKG is not oriented to identify WO-related, leg symptoms.

As previously stated, the here proposed WO detector employs a heterogeneous computing platform, implemented, and described in our previous work [6]. The developed device analyzes neuromuscular implications of PD patients in real-life scenarios, providing real-time computation in the context of PD stratification and loss of balance risk assessment by leveraging the synchronous analysis of cortical signals and EMG ones.

This paper aims to characterize an innovative set of neuromuscular features in a way that expands the potential of a device already employed in the analyses.

The breakthrough points of the proposed overall system relate to (i) the possibility of having a single device capable of monitoring various symptoms and detecting, in real-time, L-dopa-related motor fluctuations; (ii) the ability to monitor symptom asymmetry by assessing both limbs and not a single side as is the case of PKG; and (iii) providing details about the cognitive processes of movement programming and muscle status of the lower limbs in clinical support, simultaneous with detection.

## 3. Overall Architecture

**Figure 1:**
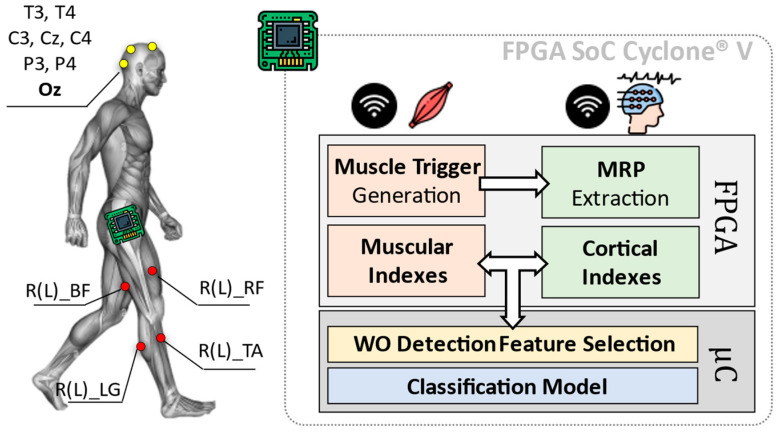
Overall architecture of the Wearing Off (WO) detector, considering an implementation of the heterogeneous computing platform by [6]. The here-proposed approach implements on a Field Programmable Gate Array (FPGA) the feature extraction phase concerning the diagnostic indexes from muscles and brain, via Movement Related Potentials (MRP) as in [6]. The classification phase is instead entrusted to the device microcontroller (μC).

A simplified overview of the here proposed system is shown in Figure 1. The employed platform consists of two main blocks: (i) a sensing and (ii) a computation system.

The first block (i.e., sensing system) consists of a wireless sensor network including electroencephalographic (EEG) signals and surface EMG.

While the computation system is in charge of analyzing EEG and EMG data from involved sensors, to extract useful cortical and muscular indexes. These indexes are subjected to a statistic-based feature selection step, which is optimized for the identification of the WO phenomenon (WO Detection Feature Selection in Figure 1). Resulting features are, thus, used to train a classification model designed to discriminate between the ON and OFF phases of the L-dopa treatment.

### 3.1. Sensing System

Eight cortical sites from a 32-channels wireless EEG headset (g.Nautilus Research by g.Tec medical engineering GmBH, Austria) have been monitored: T_3_, C_3_, C_z_, C_4_, T_4_, P_3_, P_4_ (10–20 system notation). O_z_, AFz and A2 are used for noise reduction, as reference and ground, respectively.

Muscular signals are gathered through eight wireless nodes distributed all over the lower limbs (MiniWave by Cometa System, Italy). Sensors are placed bilaterally (four per leg) on Lateral Gastrocnemius (R(L)_LG in Figure 1), Tibialis Anterior (R(L)_TA in Figure 1), Bicep Femoris (R(L)_BF in Figure 1) and Rectus Femoris (R(L)_RF in Figure 1). EEG and EMG data have been acquired at a sampling frequency f_s_ = 500 Hz with 24 bits and 16 bits of resolution, respectively.

Gathered data are sent via Bluetooth to the computation system, which is realized via the platform proposed in [6] and based on the FPGA SoC Cyclone^®^ V architecture by Intel (FPGA Cyclone^®^ V + ARM Cortex-A9 Dual-core).

### 3.2. Muscular and Cortical Indexes Extraction

The computation system is in charge of analyzing EEG and EMG data, extracting some useful cortical and muscular indexes that can be successfully used to characterize gait, PD stratification and losses of balance according to [6].

The indexes extraction runs on the FPGA and consists of two parallel processing branches. The EMG branch works in two steps: (i) Muscle Trigger Generation and (ii) Muscular Indexes extraction. During the first step (i.e., Muscle Trigger Generation), a dynamic threshold approach is used to convert the 16-bits EMG signals into 1-bit equivalent ones. The resulting binary signals (triggers in the following) are 0 when the related muscles are relaxed, while become 1 when they are contracted. More details about the dynamic thresholding method and its FPGA implementation are available at [6,14].

The binary representation of the muscular signals permits the computation of the following muscular indexes:

- *Stride time.* The time between two-foot plant strikes (Initial Simple Support in Stance Phase) of the same leg. The parameter is expressed in milliseconds (resolution 2 ms).
- *Contraction and Relaxation times.* Contraction/relaxation duration in milliseconds (resolution 2 ms). Data are extracted at the end of the stride to complete a gait cycle.
- *Duty cycle (DC).* The ratio between single muscle contraction time and stride time.
- *Co-contraction time*. Time of parallel contraction of agonist and antagonist muscle (resolution 2 ms).

The EEG branch is normally “inactive” and operates as a First-In-First-Out (FIFO) buffer, collecting the last ~500 ms of EEG data (256 samples @ 500 Hz). Once one of the lateral Gastrocnemii contracts (Initial Simple Support in Stance Phase), leading the related trigger to 1, the EEG branch activates the computation. It consists of the extraction of some indexes related to Movement-related Potentials (MRPs).

Roughly, MRPs are cortical potentials actively involved in the cerebral preparation of voluntary movements. Typically, they are detectable within specific frequency bands starting from 1 s before the movement actuation.

According to related experimental studies [21,22,23,24] and the platform settings [6], three MRPs have been assessed:

- *Bereitschafts potential (BP).* It presents as a positive component that peaks at 100–200 ms before the onset of movement. It is assessed in the frequency band ranges between 2 and 5 Hz.
- *μ-rhythm*. Detectable in a frequency band between 9 and 11 Hz and 400–500 ms before performing a motor action. The μ-rhythm suppresses when movement onset occurs.
- *β-rhythm*. This rhythm reveals in the frequency range of 12–30 Hz.

The cortical features are extracted by means of a Short-Term Fourier Transform (STFT) approach and extracting the maximum spectral content in the bands of interest. More details about the STFT and MRPs extraction FPGA implementation are available at [6].

### 3.3. Statistical Significance–Based Feature Selection

**Measurement Sessions and Protocol.** The above-described muscular and cortical features have been collected from three out of five randomly selected subjects, which are involved in the measurement sessions. This subset of subjects is identified as Training Set in Section 4.1, while data from the two not selected ones are used to realizing the Testing Set.

All involved subjects underwent two measurement sessions. Each measurement session consists of a standardized clinical protocol from the unified PD rating scale (UPDRS) guidelines [25], which is easily transferrable to daily-life scenarios: the 10-m walk. The test consists of asking subjects to walk for a distance of 10 m, adopting a comfortable walking speed.

**Statistical Sample Extraction.** One measurement session is carried out ~30 min before the planned L-dopa administration (>4h from the last administration), during a recognized OFF phase. In the following, we will refer to this session as *Pre L-dopa*. The second measurements session is carried out ~1 h after the planned administration, during a recognized ON phase. Similarly, in the following, we will refer to this session as *Post L-dopa*.

The two samples *Pre* and *Post L-dopa* are analyzed via paired sample *t*-test to identify the statistical significance of the involved features (α = 0.05).

Table 1 reports the mean values of both populations considering all the subjects in the Training Set and the related *p*-values.

Paired t-test on data from different subjects showed that the most significant features, in a generalized WO detection context, are all and only the muscular ones. No relevant variations have been recorded in cortical features.

**Figure 2:**
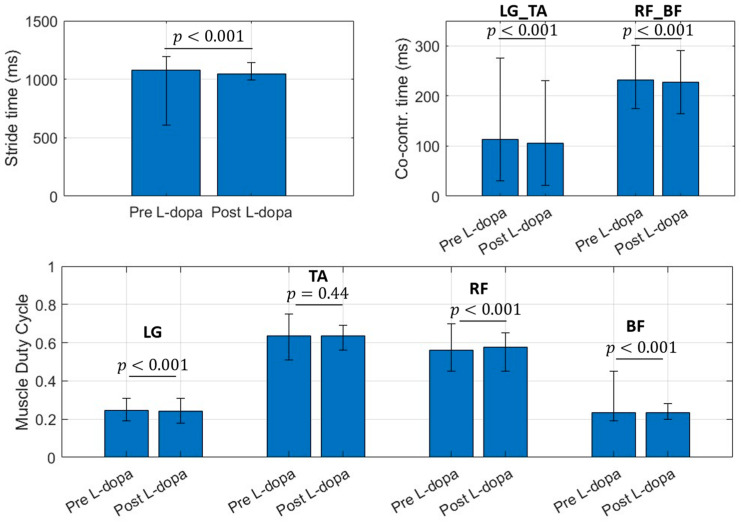
Bar chart for selected muscular features. Bar values identify the mean value for the considered parameter. Error bar limits represent the 95th percentile (upper bound) and 5th percentile (lower bound). LG_TA is the co-contraction time (ms) between Lateral Gatrocnemius (LG) and Tibialis Anterior (TA), RF_BF identifies the co-contraction time (ms) between Rectus Femoris (RF) and Bicep Femoris (BF) regardless the involved side.

For this reason, only the EMG branch can be kept active for the specific application, with a subsequent reduction in the power consumption of the device. Figure 2 shows a bar plot (with error bars) concerning the selected muscular features.

### 3.4. Classification Model

Starting from the selected muscular features, eight types of classifiers have been offline trained and employed for onboard inference via the dedicated microcontroller.

Specifically, four both shallow learning approaches and neural networks compatible with the μC resources have been analyzed. Table 2 reports the analyzed classification model.

The first four models proposed in Table 2 were trained via MATLAB^®^ 2021b and coded via a dedicated library in C language.

The best neural network (NN) models have been identified by KerasTuner [26], trained via Keras with Tensorflow backend, and C-coded exploiting X-CUBE.AI by STMicroelectronics. The tuning ranges/values of the NN parameters set in KerasTuner are reported in the following:

- *Tuner:* Hyperband [27]
- *NN Type:* Fully Connected Neural Network
- *Number of Layers (excluding the output):* 1–3
- *Number of units/layer:* 8, 16, 32, 64 (pow of 2 for parallel optimization)
- *Activation function:* Rectified Linear Unit (ReLU), Scaled Exponential Linear (SeLU), Tanh
- *Objective:* Average Validation Loss (k-fold Validation with k = 4) → Loss function: Binary Crossentropy
- *Compilation setting—Optimizer:* Nadam [28], RMSProp

The four NN models extracted via KerasTuner have been considered for the microcontroller implementation and reported in Table 2.

The optimization ranges of parameters for both shallow and deep learning approaches consider design constraints on the resources of the target microcontroller.

## 4. Results

### 4.1. Datasets

Data used for the following computations refer to the measurement sessions carried out in the context of our previous works [6,29]. Overall, five subjects (Mean Age: 71.4, M:F = 5:0) were enrolled for the study. Three of them were classified as mild PD (3rd Hoen and Yahr stage), and n = 2 subjects were classified as severe PD (4th stage of Hoen and Yahr stage). The patients were characterized in this way by specialized medical staff who supervised the standard protocol. The patients received a PD diagnosis 9–11 years before the measurement sessions. They started L-dopa treatment contextually [28]. Data from three out of the five involved subjects have been used to realize the *Training Set*, while data from the remaining two subjects constituted the *Testing Set*.

As previously stated, the measurement session consists of a standardized clinical protocol from UPDRS guidelines [17]: the 10-m walk. The protocol is carried out as prescribed by UPDRS III by the sections: III.10 March, III.14 Bradykinesia, and in UPDRS-IV as IV.1 Dyskinesia time, IV.3 Motor Fluctuations. The protocol is repeated eight times collecting 150 steps per run for a total of 1200 steps per session. The steps carried out to curve the trajectories are excluded from further computation.

A detailed description of the employed datasets is reported in Table 3.

### 4.2. WO Tracker Performance

The eight classifiers, reported in Table 2, were offline trained and validated according to the procedures explained in Section 3.4. The extracted models have been instantiated on the microcontroller and tabular data from Testing Set have been streamed to the proposed system for testing purposes.

#### 4.2.1. Population Distribution

**Figure 3:**
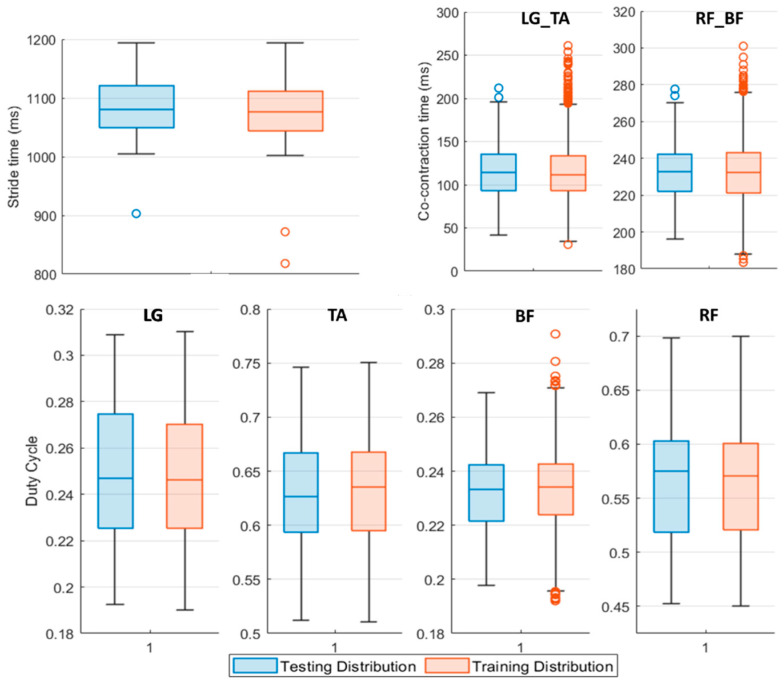
Boxplot representation of the Testing (blue boxplot) and Training (red boxplot) Set features distribution. The middle line of the boxplot represents the median value of the distribution, upper and lower boundaries of the boxplot are respectively the 75^th^ and the 25^th^ percentile of the considered sample. The upper and lower limits of the bar are the maximum and minimum adjacent, while the circles denote outliers. LG_TA identifies the co-contraction time (ms) between Lateral Gatrocnemius (LG) and Tibialis Anterior (TA), similarly, RF_BF concerns Rectus Femoris (RF) and Bicep Femoris (BF) muscles regardless of the involved side.

Figure 3 shows the input distribution of every feature composing the Testing Set with respect to the Training Set one.

Data reported in Figure 3 refer to the features population for *Pre L-dopa* measurement sessions. The 25th and 75th percentile of the testing population distribution fall within the same boundaries of the dataset used to train the classifiers, except for the LG and RF duty cycle values. On average, subjects in the *Pre L-dopa* session show a stride time of 1092 ± 72.01 ms, and a Co-contraction time on LG-TA and RF-BF of 113.5 ± 32.12 ms and 232.5 ± 50.04 ms, respectively. The duty cycle on LG is 24.81 ± 3.7 %, on TA 56.33 ± 5.44 %, on RF 63.48 ± 5.49 %, and 23.32 ± 2.42 % on BF. During the *Post L-dopa* session, stride time decreased to 1044 ± 34.49 ms, and co-contraction times on LG-TA and RF-BF also reduced to 96.2 ± 29.19 ms and 202.14 ± 17 ms, respectively.

#### 4.2.2. Performance Metrics

A test session per each analyzed classifier in Table 2 has been carried out. For every model, a confusion matrix, the Receiver operating characteristic (ROC) curve, the Area Under Curve (AUC) and four standard metrics derived according to Equations (1)–(4) are extracted:

(1) $$Accuracy=\frac{tp+tn}{tp+tn+fp+fn}\cdot 100$$

(2) $$Recall=\frac{tp}{tp+fn}\cdot 100$$

(3) $$Precision=\frac{tp}{tp+fp}\cdot 100$$

(4) $$F1-score=\frac{2\cdot tp}{2\cdot tp+fn+fp}\cdot 100$$

where tp is the true positive parameter, tn is the true negative, fp is the false positive, fn is the false negative, considering the *Pre L-dopa* as the positive class.

**Figure 4:**
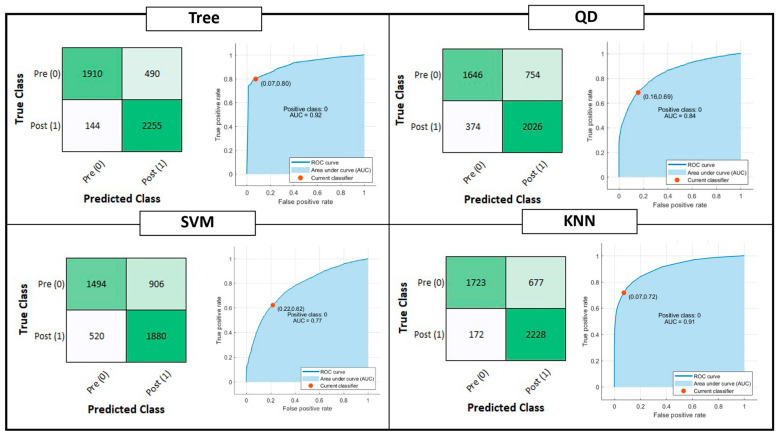
Shallow Learning Classification model performances: Confusion matrix, Receiver Operating Characteristic (ROC) curves and Area Under Curve (AUC) parameter. The positive class selected for the reported computation is the OFF phenomenon of Wearing Off (WO). The red dot on the ROC denotes the selected and analyzed classifier, minimizing the distance among the True Positive Rate (TPR) and False Positive Rate (FPR) values of the ROC curve and {FPR, TPR} = {0,1}.

**Figure 5:**
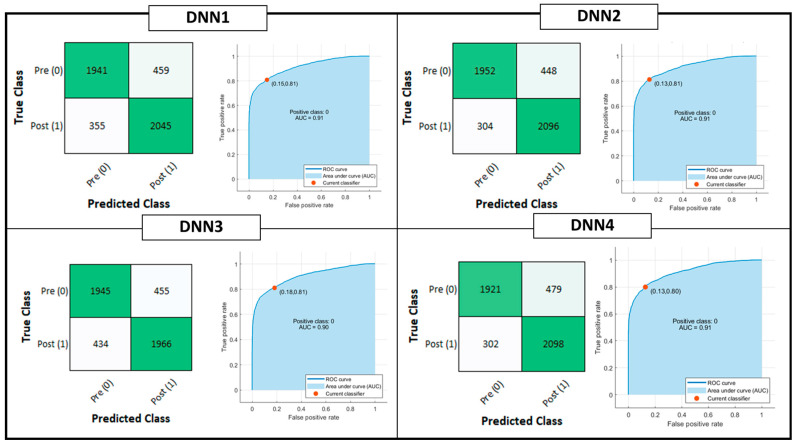
Deep Learning Classification model performances: Confusion matrix, Receiver Operating Characteristic (ROC) curves and Area Under Curve (AUC) parameter. The positive class selected for the reported computation is the OFF phenomenon of Wearing Off (WO). The red dot on the ROC denotes the selected and analyzed classifier, minimizing the distance among the True Positive Rate (TPR) and False Positive Rate (FPR) values of the ROC curve and {FPR, TPR} = {0,1}.

Figure 4 shows the confusion matrix and ROC curve for Tree, QD, SVM and KNN model classifiers considering the Testing Set. Figure 5 is, instead, dedicated to the dense NNs (DNN#) whose characteristics are proposed in Table 2. Table 4 summarizes the performance metrics for each implemented classifier. The category of classifiers that overcome the threshold of 80% for all the considered metrics is the DNN. Proposed DNNs also ensure a limited complexity in implementation and minimum resource consumption. Tree classifier also performs well, overcoming 92% in Precision metric by exploiting 129 threshold comparisons. Due to the tradeoff between implementation easiness (low memory and resources consumption) and achieved performance, DNNs are considered the best choice for embedded systems applications, and will be considered for further analysis. Tree classifier also performs well, overcoming 92% in Precision metric by exploiting 129 threshold comparisons.

Due to the tradeoff between implementation easiness (low memory and resources consumption) and achieved performance, DNNs are considered the best choice for embedding classification steps on the heterogeneous computing device microcontroller. Indeed, the NN considering the highest number of parameters is DNN#1 with only 3 hidden layers with 32 units per layer. For this reason, only DNNs are considered for further analysis.

Table 5 summarizes a comparison of the proposed system with the state-of-the-art solutions analyzed in Section 2, in terms of classification performance. Technological proposals for WO detection outlined in [13,14] have been excluded during the comparison, because they do not implement a classification step for the proposed features. Moreover, no details about the number of units per each of the 15 hidden layers composing the NN are provided in [15]. On average, the proposed solution performance is found to be perfectly in line with those related to the state of the art.

However, it should be pointed out that, although the results are interesting and promising, the metrics are not directly comparable. In fact, the system proposed in this paper provides a generalized classification model (based on data collection from 3 subjects and tested on 2 different subjects), rather than a subjective one. The use of a generalized model rather than a user-tailored approach leads to an underestimation of the performance of the proposed system, which remains competitive, allowing its wider application with a reduction of relative training time.

### 4.3. WO Tracker Timing

The here proposed system, supplied with the DNN4 has been tested in terms of prediction timing. In this frame, the FPGA-implemented EMG trigger extraction block requires 40 ms to activate the trigger starting from the actual contraction [6], leading to a static delay in index assessment. When the stride is formally completed, the Muscular indexes block is enabled. It requires 0.2 ms to extract and pass features from programmable logic (FPGA) to the processing system (microcontroller) that implements the classifier. Once the features are available at DNN1, it needs 2.8 ms (@ 8 MHz clock) to complete inference, resulting in a total timing of about 43 ms after the completion of the step. If DNN2 is employed the inference time results to be 1.57 ms (41.8 ms total). With DNN3 and DNN4 inference times are 3.12 ms (43.32 ms total) and ~350 μs (40.55 ms total), respectively.

### 4.4. WO Tracker Complexity

In this section, the proposed NNs are analyzed in terms of implementation complexity, by extracting three main metrics: RAM, ROM/Flash, Multiply and ACCumulate (MACC). RAM indicates the size (in bytes) of the expected R/W memory used to store the intermediate inference computing values and the dedicated library, ROM/Flash indicates the size of the generated read-only memory to store weight and bias parameters, while MACC operations define the functional complexity of the imported NN. Table 6 summarizes the implementation complexity metrics for implemented DNNs.

Considering the complexity, accuracy and timing parameters, the best choice seems to be DNN4. It allows, on average, to speed up the inference system of a factor 7, reducing the flash occupancy of the 7.6 factor, without affecting accuracy on Testing Set (−0.61% w.r.t. DNN2).

### 4.5. WO Tracker Power Consumption

In terms of power consumption, the proposed system must be divided into two subsystems: the sensors set and the computing unit. Consider the first one, the wireless EMG sensors employ a 3.7 V lithium battery of 28 × 20 × 12 mm and absorb 40 mW with a sampling rate of 2 kHz and apparent radiated power (ARP) of 0.45 mW at 2.4 MHz. In a continuous-mode operation, the EMG nodes can last up to 12 h. The EEG uses a Lithium-Ion Prismatic 523,450 3.7 V and presents a power consumption of 500 mW. A typical continuous acquisition reaches about 8 h. In the specific application, this last module is not employed. The PowerPlay power analyzer tool used to provide the power consumption values related to the computing unit reported that the overall implementation consumes 519.57 mW (453.24 mW if the EEG branch is turned off) without heat sink with still air, of which 416.64 mW is a core static thermal power dissipation. The power dissipation caused by signal transitions (dynamically dissipated) is estimated as 88.89 mW with the EEG branch turned on. Turning off the EEG branch, as per our case, the overall dynamic consumption reduces to 28.60 mW mainly divided into 3.60 mW for the I/O, 1.04 mW for the register cells, and 0.04 mW, and finally, 11.55 mW for the PLL unit consumption. The remaining power consumption quote is due to microcontroller computation.

## 5. Conclusions

To date, L-dopa treatment is still the most used dopamine replacement therapy for the treatment of PD. However, excessive and extensive use of L-dopa leads, within 3–5 years, to the onset of the WO phenomenon. This phenomenon, although widespread among patients with PD, is often undiagnosed. In addition, the onset of symptoms between one administration and another does not allow the patient a continuously good quality of life. In the personalized medicine era, technological support to allow clinicians a release of personalized treatment plans based on WO phenomenon detection is needed. In this context, this paper presented a wearable WO tracker that exploits the feature extraction capabilities of a preexisting embedded platform, which is born for PD stratification and postural assessment. The proposed technological solution analyzes cortical and muscular data wirelessly gathered by an EEG headset and a set of eight surface EMG sensors to identify the neuromuscular involvement during the ON/OFF phase typical of WO fluctuations. The proposed architecture, implemented on a heterogeneous computing platform, statistically analyzes neural and muscular features through a statistical significance-based approach, in order to find a highly descriptive set of features employable for the classification step. An analysis concerning the best classification model has been conducted on four shallow learning and four deep learning approaches. Each model has been assessed in terms of accuracy performance, inference timing, and implementation ease. The selected classifier consists of a 1-layer dense NN. The hidden layer embeds 32 units and a ReLU activation function. With 311 MACC operations (~350 μs/inference), 1.05 kB of ROM, and 3.13 kB of RAM, the proposed NN is able to reach an accuracy of 83.72%, with a precision on the OFF state recognition of 86.41 % on a Testing Set based on two subjects. The proposed embedded platform is demonstrated to be easily employed as a daily life monitoring system to record WO-related symptom fluctuations. These system features are useful to support physicians in providing continuous and progressive adaption of L-dopa treatment plans.

Although the experimental results are promising in terms of performance and ease of implementation of the WO detector, some implementation details remain to be investigated.

The proposed system has been optimized and evaluated only on standardized walks and never interspersed with ordinary life actions that may introduce high variability in the neuromuscular patterns. Therefore, the system should be also verified in an ordinary life scenario introducing a dedicated rejection strategy, if needed.

In addition, to improve the performance of the proposed WO detection system, data from IMU sensors, integrated into the EMGs used for the application, could be exploited. However, this step must consider the data stream to be analyzed, which adversely affects the memory resources of the employed device.

## Figures and Tables

**Table 1:** Statistical significance analysis for extracted cortico-muscular features.

Feature	μPre	μPost	*p*
Stride Time (ms)	1081.09	1044.64	<0.001
Co-Con. LG-TA (ms) ^1^	113.74	106.16	<0.001
Co-Con. BF-RF (ms) ^1^	232.51	228.02	<0.001
DC LG (%)	24.78	24.34	<0.001
DC TA (%)	63.48	63.49	0.44
DC BF (%)	23.31	23.54	<0.001
DC RF (%)	56.29	57.49	<0.001
BP | μ | β T_4_ (dBμ) ^2^	61.97 | 47.97 | 40.50	62.00 | 48.03 | 40.49	0.26 | 0.07 | 0.31
BP | μ | β T_3_ (dBμ) ^2^	60.49 | 48.02 | 40.00	60.47 | 48.04 | 39.99	0.21 | 0.34 | 0.32
BP | μ | β C_4_ (dBμ) ^2^	59.50 | 46.99 | 40.97	59.47 | 47.01 | 41.00	0.20 | 0.41 | 0.25
BP | μ | β C_3_ (dBμ) ^2^	61.51 | 48.96 | 42.51	61.50 | 49.02 | 42.51	0.34 | 0.08 | 0.49
BP | μ | β C_z_ (dBμ) ^2^	62.50 | 49.52 | 37.49	62.51 | 49.53 | 37.49	0.36 | 0.40 | 0.49
BP | μ | β P_4_ (dBμ) ^2^	63.02 | 48.51 | 44.03	63.01 | 48.47 | 44.02	0.38 | 0.16 | 0.40
BP | μ | β P_3_ (dBμ) ^2^	63.02 | 48.47 | 43.98	63.00 | 48.48 | 43.97	0.36 | 0.37 | 0.39

^1^ Co-contraction from left and right limbs are considered together neglecting the possible unilateral involvement of PD. ^2^ The parameter is calculated according to [6] as 20 · log(max(MRP[V])/1μV)).

**Table 2:** Analyzed Classification Models.

Classifier Model	Note	Acronym
Tree	Number of split *: 129, Split criterion *: Gini’ s diversity index	n.a.
Discriminant	Discriminant Type *: Quadratic	QD
Support Vector Machine	Kernel Function: Linear ^†^	SVM
k-Nearest Neighbors	Number of neighbors *: 21 Distance metric *: City block Distance weight *: Equal	KNN
NN #1	Number of Layers: 3 Number of units/layer 1,2,3: 32 Activation function layer 1,2,3: ReLU Input Layer: Batch Normalization Output Layer: 1 unit + Sigmoid	DNN1
NN #2	Number of Layers: 2 Number of units/layer 1,2: 32 Activation function layer 1,2: ReLU Input Layer: Batch Normalization Output Layer: 1 unit + Sigmoid	DNN2
NN #3	Number of Layers: 2 Number of units/layer 1,2,3: 32 Activation function layer 2,3: Tanh Activation function layer 2,3: ReLU Input Layer: Batch Normalization Output Layer: 1 unit + Sigmoid	DNN3
NN #4	Number of Layers: 1 Number of units/layer: 32 Activation function: ReLU Optimizer: RMSProp Input Layer: Batch Normalization Output Layer: 1 unit + Sigmoid	DNN4

* Parameter optimized via Hyperparameter Search Engine provided by MATLAB^®^ 2021b; ^†^ MATLAB^®^ 2021b default settings.

**Table 3:** Training and Test Datasets description and distribution.

Dataset	Description	Observations
Training Set	n = 2 randomly selected patients mild PD + n = 1 randomly selected patient with severe PD	*Pre L-dopa:* 3600 steps *Post L-dopa:* 3600 steps
Testing Set	n = 1 randomly selected patient mild PD + n = 1 randomly selected patient with severe PD	*Pre L-dopa:* 2400 steps *Post L-dopa:* 2400 steps

**Table 4:** Performance metrics for implemented classification models.

Classifier	Accuracy	Recall	Precision	F1-score	AUC
Tree *	**86.79**	79.58	**92.98**	**85.76**	**0.92**
QD *	76.50	68.58	**81.48**	74.48	0.84
SVM *	70.29	62.25	74.18	67.69	0.77
KNN *	**82.31**	71.79	**90.92**	**80.23**	**0.91**
DNN1	**83.04**	**80.87**	**84.53**	**82.66**	**0.91**
DNN2	**84.33**	**81.33**	**86.52**	**83.84**	**0.91**
DNN3	**81.48**	**81.04**	**81.76**	**81.40**	**0.90**
DNN4	**83.72**	**80.04**	**86.41**	**83.11**	**0.91**

* Considered classifiers have been trained via Matlab^®^ 2021b considering a cross-validation with k = 4 (default).

**Table 5:** Performance metrics: State-of-the-Art Comparison.

Ref., Year	Accuracy	Sensitivity (Recall)	Specificity
[15], 2021	RGLM: 72.4 NN: 80.2 RF: 86.8	RGLM: 88 NN: 93 RF: 97	RGLM: 78 NN: 81 RF: 93
[16], 2020	Best: 83.56	Best: 78.51	Best: 92.02
[17], 2022	77.04	77.04	n.a.
This work (Tree)	86.79	79.58	93.99
This work (DNN1)	83.04	80.87	85.20
This work (DNN2)	84.33	81.33	87.33
This work (DNN3)	81.48	81.04	81.92
This work (DNN4)	83.72	80.04	87.41

**Table 6:** Implementation complexity metrics for implemented DNNs.

Classifier	RAM (kB)	Flash (kB)	MACC
DNN1	3.13	9.30	2487
DNN2	3.13	5.18	1399
DNN3	3.13	9.30	2775
DNN4	3.13	1.05	311

## Data Availability

More details concerning analyzed dataset are provided in [6,29].
